# Monitoring of radioactive contamination in Polish surface waters in 2012–2013

**DOI:** 10.1007/s10967-014-3728-3

**Published:** 2014-11-02

**Authors:** M. Suplińska, M. Kardaś, B. Rubel, A. Fulara, A. Adamczyk

**Affiliations:** Central Laboratory for Radiological Protection, 03-194 Warsaw, Poland

**Keywords:** Surface water, Contamination, ^90^Sr, ^137^Cs

## Abstract

The ^90^Sr and ^137^Cs contamination in Polish surface waters has been monitoring since 1994. Surface water samples from six lakes and the Vistula and Oder Rivers were collected in spring and autumn 2012 and 2013. The mean ^90^Sr and ^137^Cs concentrations were 3.92 ± 0.40 and 4.49 ± 2.00 mBq L^−1^, respectively. Correlations were identified between the radionuclide concentrations and meteorological conditions and the original fallout distribution from the Chernobyl disaster. The annual average radionuclide concentrations were not significantly different from the concentrations found between 1994 and 2011. The ^137^Cs and ^90^Sr concentrations have been decreasing only slowly.

## Introduction

Artificial radioactive isotopes are present in the environment because of human activities, and these isotopes include (a) fission products generated in nuclear explosions in the atmosphere, which were performed by several countries until 1980, and (b) the products of the Chernobyl nuclear reactor accident in April 1986. The radionuclides ^137^Cs and ^90^Sr are present in the environment at higher concentrations than other artificial radionuclides. The systematic monitoring of radionuclides in the Polish environment has been performed by the Central Laboratory for Radiological Protection (CLOR) since the early 1960s. For two decades the monitoring study was focused on radionuclide contamination in surface waters (the Baltic Sea and Rivers), tap water, soil, air, agricultural products, and foodstuffs that was predominantly caused by nuclear explosions in the atmosphere. Short- and long-lived radionuclides were measured, and the main indicators of long-term pollution related to weapons were ^90^Sr (*t*_1/2_ = 28.8 year) and ^137^Cs (*t*_1/2_ = 30.17 year). The extent of the monitoring study was increased dramatically after the Chernobyl disaster.

The CLOR and the network of stations that form the service for the measurement of radioactive contamination has been required to perform high-frequency analyses of the total β-particle activities in surface water (rivers and lakes) and tap water samples [1]. The CLOR started a systematic study of radioactive contamination in water and bottom sediment samples from Polish rivers and lakes in 1994. At present, six lakes in different areas and the two main river systems in Poland, the Vistula River and two of its tributaries (the Bug and Narew Rivers) and the Oder River and its main eastern tributary (the Warta River), are regularly monitored. The activity concentrations of ^137^Cs and ^90^Sr are measured in the water samples, and the ^137^Cs and ^239,240^Pu activity concentrations are determined in the bottom sediment samples. Here, we present the results of the analyses of the water samples for ^137^Cs and ^90^Sr.

## Experimental

### Description of sampling sites

The Vistula River is the longest river in Poland, with a total length of 1,022 km. The part of the Vistula River drainage basin that is in Poland measures 168,868 km^2^. The main tributaries of the Vistula River come from the east, and are the Bug River (590 km of which is within Poland) and the Narew River (443 km of which is within Poland). The sampling points on the Vistula River were in the upper, middle, and lower parts, in Kraków, Annopol, Warsaw, Płock, and Kiezmark. The sampling points on the Narew and Bug Rivers were in Pułtusk and Wyszków, respectively.

The Oder River is 840 km long, and 726 km of the river is within Poland and along the Polish–German border. The part of the Oder River drainage basin that is within Poland measures 106,043 km^2^. The main tributary of the Oder River comes from the east, and is the Warta River, which is 795 km long. The sampling points on the Oder River were at Chałupki, Wrocław, Głogów, and Krajnik, and the sampling point on the Warta River was at Poznań.

**Fig. 1:**
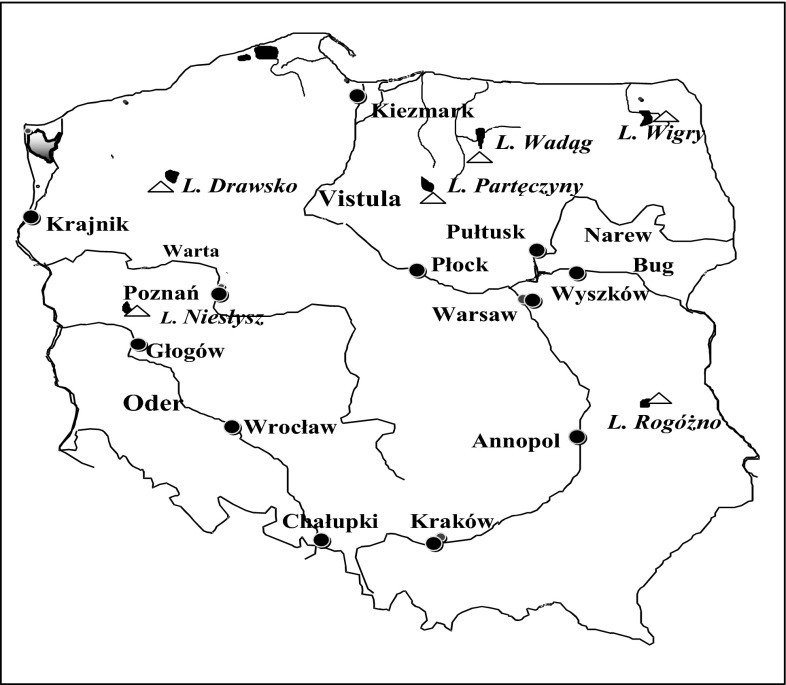
Sampling sites (*circles* indicate river sampling cites and* triangles* indicate lake sampling sites)

Six lakes, each located in a different part of Poland, were selected for the water monitoring program. The lakes were Drawsko Lake (in the West Pomeranian District), Niesłysz Lake (in the Lubuskie District), Wadąg and Partęczyny Lakes (in the Masurian District), Wigry Lake (in the Podlaskie District), and Rogóźno Lake (in the Lubelskie District). The largest of the lakes were Wigry Lake (which has an area of 21.2 km^2^ and a depth of 73 m) and Drawsko Lake (which has an area of 17.8 km^2^ and a depth of 79.7 m), and Rogóźno Lake (which has an area of 0.57 km^2^ and a depth of 25.5 m) was the smallest lake. The locations of the river and lake sampling sites are shown in Fig. 1.

### Sample preparation

Water samples were collected in the spring and autumn each year. The water samples were 20 L, and they were collected from the main stream of each river and from a platform extending from the shore into each lake. The water samples were put into polyethylene containers and acidified with HNO_3_. In the laboratory, each water sample was evaporated to 450 mL and filtered through a hard filter paper. The filter was then ashed in an oven at 450 °C, and the ash was dissolved in nitric acid and combined with the filtrate. Finally, each water sample was evaporated to 250 mL and the ^137^Cs and ^90^Sr concentrations in the sample were determined.

### Analytical method

The ^137^Cs in a sample was separated from the solution by filtering the sample through a ^137^Cs-selective bed of ammonium molybdophosphate in a radiochemical funnel [2]. The β-particle activity in the ammonium molybdophosphate bed was then measured using a low-level beta GM multicounter system (DTU Nutech, Roskilde, Denmark).

After the cesium was removed, the ^90^Sr concentration in the filtrate was determined using the Volchok method [3] with some modifications. The ^90^Sr concentration was determined from the β-radiation emitted by ^90^Y once equilibrium was reached in the ^90^Sr–^90^Y system. The same conditions were used for the ^90^Sr measurements as were used for the ^137^Cs measurements. The accuracy of each of the analytical methods was verified in national and international comparison exercises.

## Results and discussion

The ^137^Cs and ^90^Sr concentrations in the water samples from the Vistula and Oder Rivers and their tributaries and from the lakes are shown in Table 1. The samples were collected in the same periods (spring and autumn) in 2012 and 2013, although the atmospheric and hydrologic conditions were different in the different years. There was a drought in 2012, and the spring samples were collected when there were low or very low water levels. This may have affected the overall results.

**Table 1:** ^137^Cs and ^90^Sr activity concentrations (mBq L^−1^) in water samples from the Vistula and Oder Rivers, their tributaries, and six lakes in 2012 and 2013

River/lake	Sampling point	^137^Cs activity concentration (mBq L^−1^)				^90^Sr activity concentration (mBq L^−1^)			
		2012		2013		2012		2013	
		Spring	Autumn	Spring	Autumn	Spring	Autumn	Spring	Autumn
Vistula River and its tributaries									
Vistula	Kraków-Tyniec	7.05 ± 0.43	3.40 ± 0.29	5.65 ± 0.63	4.18 ± 0.49	4.29 ± 0.33	7.24 ± 0.33	4.46 ± 0.87	2.56 ± 0.52
	Annopol	12.33 ± 0.58	3.67 ± 0.30	2.63 ± 0.37	3.59 ± 0.43	5.32 ± 0.36	4.18 ± 0.23	4.38 ± 0.81	3.25 ± 0.66
	Warsaw	9.71 ± 0.49	1.25 ± 0.18	2.97 ± 0.37	1.92 ± 0.18	9.61 ± 0.51	2.18 ± 0.16	4.62 ± 0.90	3.41 ± 0.70
	Płock	2.64 ± 0.24	3.68 ± 0.29	2.99 ± 0.37	2.82 ± 0.26	4.00 ± 0.32	3.26 ± 0.27	5.05 ± 0.96	1.96 ± 0.40
	Kiezmark	3.04 ± 0.27	2.07 ± 0.21	3.27 ± 0.40	2.64 ± 0.35	3.34 ± 0.27	2.71 ± 0.17	5.71 ± 1.20	1.59 ± 0.33
Narew	Pułtusk	2.48 ± 0.24	1.29 ± 0.23	2.77 ± 0.35	2.43 ± 0.31	4.39 ± 0.38	1.59 ± 0.13	3.61 ± 0.69	2.46 ± 0.50
Bug	Wyszków	2.13 ± 0.22	2.33 ± 0.34	2.00 ± 0.27	4.30 ± 0.49	5.08 ± 0.37	3.32 ± 0.19	11.59 ± 2.18	1.56 ± 0.32
Oder River and its tributaries									
Oder	Chałupki	40.72 ± 1.04	4.95 ± 0.35	1.66 ± 0.24	7.25 ± 0.79	3.26 ± 0.28	4.86 ± 0.27	4.88 ± 0.88	3.01 ± 0.61
	Wrocław	6.49 ± 0.41	6.00 ± 0.40	5.16 ± 0.58	5.30 ± 0.59	3.72 ± 0.30	4.03 ± 0.22	4.60 ± 0.83	3.70 ± 0.76
	Głogów	3.30 ± 0.28	4.23 ± 0.32	4.42 ± 0.41	4.22 ± 0.49	2.15 ± 0.25	4.90 ± 0.24	4.65 ± 0.88	2.54 ± 0.52
	Krajnik	3.73 ± 0.31	1.98 ± 0.22	2.41 ± 0.16	2.03 ± 0.15	2.65 ± 0.24	2.22 ± 0.16	4.83 ± 0.87	3.45 ± 0.70
Warta	Poznań	10.96 ± 0.54	2.41 ± 0.17	1.77 ± 0.25	2.18 ± 0.29	4.39 ± 0.35	3.18 ± 0.19	6.00 ± 1.16	6.22 ± 1.27
Lakes									
Wigry	Stary Folwark	2.86 ± 0.26	1.97 ± 0.21	2.33 ± 0.30	2.94 ± 0.36	2.76 ± 0.26	2.86 ± 0.28	2.52 ± 0.48	1.42 ± 0.29
Wadąg	Myki	2.54 ± 0.24	1.59 ± 0.18	1.50 ± 0.22	1.88 ± 0.26	2.79 ± 0.27	3.12 ± 0.19	2.65 ± 0.57	2.26 ± 0.46
Wielkie Partęczyny	Partęczyny	4.89 ± 0.34	4.34 ± 0.32	2.70 ± 0.34	1.06 ± 0.17	2.20 ± 0.23	3.18 ± 0.30	2.16 ± 0.45	1.69 ± 0.34
Drawsko	Stare Drawsko	4.78 ± 0.34	7.25 ± 0.43	2.81 ± 0.30	0.76 ± 0.14	5.36 ± 0.39	4.34 ± 0.21	3.67 ± 0.72	4.68 ± 0.96
Niesłysz	Niesulice	3.60 ± 0.29	3.80 ± 0.31	5.63 ± 0.56	2.67 ± 0.34	2.49 ± 0.25	1.60 ± 0.13	2.49 ± 0.49	1.35 ± 0.28
Rogóźno	Rogóźno	9.07 ± 0.50	5.38 ± 0.26	5.88 ± 0.65	5.30 ± 0.59	6.19 ± 0.41	9.10 ± 0.36	8.36 ± 1.63	7.56 ± 1.55

The highest ^137^Cs concentration (12.33 mBq L^−1^) in the water samples from the Vistula River was found in the sample that was collected in spring 2012 in Annopol. Relatively high ^137^Cs concentrations were also found in the samples collected from Warsaw (9.71 mBq L^−1^) and Kraków (7.05 mBq L^−1^) in spring 2012. The concentrations in the samples from Kraków and Annopol collected in autumn 2012 (3.40 mBq L^−1^ in Kraków and 3.67 mBq L^−1^ in Annopol) were lower by a factor of two to three than the concentrations in the samples collected in the spring. The concentrations in the samples from Warsaw were lower by a factor of eight (at 1.25 mBq L^−1^) in the autumn than in the spring. The differences between the concentrations found in the river water samples collected in the spring and autumn in 2013 were fairly small. Relatively high ^137^Cs concentrations were only found in the river water samples from Kraków in 2013 (5.65 mBq L^−1^ in the spring and 4.18 mBq L^−1^ in the autumn). The water samples collected from the lower Vistula River in 2012 and 2013 were characterized by low ^137^Cs concentrations and only small variations in the concentrations, and this agrees with data for previous years [4]. The mean ^137^Cs concentrations in the samples collected from the Vistula River in Płock and Kiezmark in 2012 and 2013 were 3.03 ± 0.45 and 2.76 ± 0.53 mBq L^−1^, respectively.

The ^137^Cs concentrations found in the water samples from the Narew and Bug Rivers were generally lower (2–3 mBq L^−1^ in both rivers) than the concentrations that were found in the samples from the Vistula River in the same sampling periods. The only exception to this being in the autumn of 2013, at which time the sample collected from the Bug River contained a ^137^Cs concentration of 4.3 mBq L^−1^.

The ^137^Cs concentrations were distinctly different in the samples from the different sampling points on the Oder River, but the ^137^Cs concentrations were quite consistent over the entire sampling period at each sample site. The mean ^137^Cs concentrations in the samples collected in Wrocław, Głogów, and Krajnik were 5.74 ± 0.62, 4.04 ± 0.50, and 2.54 ± 0.82 mBq L^−1^, respectively, and it can be seen that the concentration clearly decreased moving downstream. The largest temporal differences in the ^137^Cs concentrations were found at the Chałupki site, where the ^137^Cs concentrations were 40.72 mBq L^−1^ in spring 2012 and 1.66 mBq L^−1^ in spring 2013. The Chałupki site is in the region of Poland that suffered the most contamination from the Chernobyl accident. Local meteorological conditions could, therefore, have affected the ^137^Cs concentrations found in the samples that we analyzed. Similar to the difference in the ^137^Cs concentrations in the water collected in Chałupki, the ^137^Cs concentrations in the samples collected from the Warta River were five times higher in spring 2012 than in spring 2013.

The ^137^Cs concentrations in the samples from the lakes varied strongly between sample sites and between sampling periods. The highest ^137^Cs concentration was found in water from the small Rogóźno Lake, and significantly lower concentrations were found in the samples from the larger and deeper lakes (Wadąg and Wigry Lakes) in northeastern Poland. The mean ^137^Cs concentrations found in the samples collected in 2012 and 2013 were 6.41 ± 1.79 mBq L^−1^ for Rogóźno Lake, 1.88 ± 0.49 mBq L^−1^ for Wadąg Lake, and 2.53 ± 0.46 mBq L^−1^ for Wigry Lake. Similar differences between ^137^Cs concentrations in water samples from the lakes were found in previous studies [4, 5].

The ^90^Sr concentration in the river water samples varied less between sample sites and temporally than did the ^137^Cs concentrations. The ^90^Sr concentration ranged from 1.59 to 9.61 mBq L^−1^ over the study period, and the means (over 2012 and 2013) were 4.32 ± 1.20 mBq L^−1^ for the samples from the Vistula River, 4.02 ± 1.17 mBq L^−1^ for the samples from the tributaries of the Vistula River, 3.54 ± 0.64 mBq L^−1^ for the samples from the Oder River, and 4.39 ± 1.00 mBq L^−1^ for the samples from the tributaries of the Oder River. The highest ^90^Sr concentration in the river water samples (9.61 mBq L^−1^) was found in a sample from the Vistula River collected in Warsaw in spring 2012, but a relatively high concentration (7.24 mBq L^−1^) was found in a sample from the Vistula River collected in Kraków in autumn 2012. In 2012, the ^90^Sr concentration in the samples from the Oder (collected at Głogów) ranged from 2.15 mBq L^−1^ in the spring to 4.9 mBq L^−l^ in the autumn, and these samples were collected when the water levels were low. The ^90^Sr concentration in the sample collected in Chałupki in spring 2013 was 4.88 mBq L^−1^, and this decreased to 3.01 mBq L^−1^ in the sample collected in autumn 2013. We concluded that the mean ^90^Sr concentrations in the samples from both rivers were very similar. However, the ^90^Sr concentrations in the samples from the Warta River were somewhat more variable, and the mean ^90^Sr concentration in 2013 (6.11 mBq L^−1^) was higher than the mean ^90^Sr concentration in 2012 (3.79 mBq L^−1^).

As was the case for ^137^Cs, the ^90^Sr concentrations were higher in the samples from the small Rogóźno Lake (in southeastern Poland), ranging from 6.19 mBq L^−1^ in spring 2012 to 9.2 mBq L^−1^ in autumn 2012, than the ^90^Sr concentrations in the samples from the other lakes. The ^90^Sr concentrations in the samples from Drawsko Lake were also relatively high, ranging from 3.67 mBq L^−1^ in spring 2013 to 5.36 mBq L^−1^ in spring 2012. Lower ^90^Sr concentrations were found in Niesłysz Lake (1.35 and 1.60 mBq L^−1^ in the autumn samples and 2.49 mBq L^−1^ in the spring samples). The mean ^90^Sr concentrations in the samples from the lakes were 3.84 ± 2.14 mBq L^−1^ in 2012 and 3.40 ± 2.33 mBq L^−1^ in 2013.

As is stated in the introduction, the radioactive contamination in water was monitored using the total β-particle activity before 1986. The mean β-particle activity found in the Polish water samples that were analyzed in 1986 was 1.5 Bq L^−1^, and the highest total β-particle activity reached 417 Bq L^−1^ in May 1986, when the Chernobyl disaster occurred. The total β-particle activities found in water samples in 2001 were 24–386 mBq L^−1^. Since 1986, the ^137^Cs in Polish water samples has predominantly originated from the fallout from the Chernobyl accident, which caused severe contamination in some regions, especially in eastern and southwestern Poland. The ^137^Cs concentrations in soil show that the amount of ^137^Cs contamination in the Polish environment has decreased by a factor of 2.5 since 1988, but the geographical variations in the contamination have not changed [6]. There is still a clear relationship between the geographical location and the ^137^Cs concentrations in soil and water samples. The main source of ^137^Cs to rivers is it being washed out of the soil by water. This partly explains why there are higher ^137^Cs concentrations in the upper parts of the Vistula and Oder Rivers, which are in southern Poland, than in the rivers near to their Baltic Sea estuaries. There was a correlation between the locations of the lakes and the radioactive fallout distribution. A particular case is Rogóźno Lake (in eastern Poland) which has no inflowing or outflowing rivers, so no water exchange can occur, and the only water supplies to the lake are springs and precipitation. All of the other lakes that were studied belong to water systems that include rivers.

**Fig. 2:**
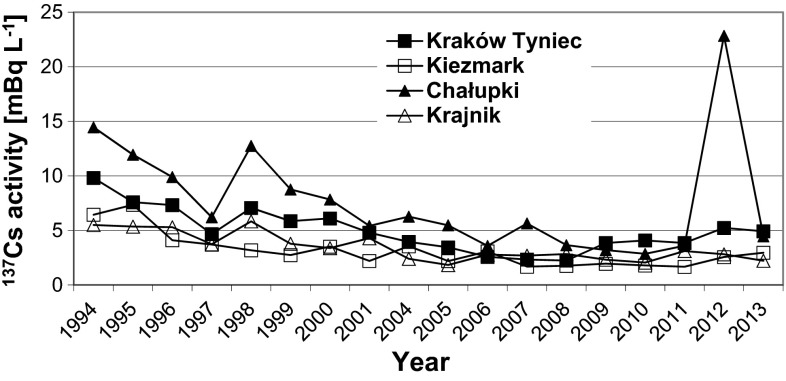
Annual average ^137^Cs activity concentrations in water samples from four sampling sites

The impact of contamination caused by the Chernobyl disaster and meteorological conditions on the ^137^Cs concentrations in the river water samples can be identified by inspecting the long-term trends in the ^137^Cs concentrations. The plots shown in Fig. 2 (see Table 1 for the detailed results) represent the mean annual ^137^Cs concentrations over 20 y (1994–2013) at selected sampling points in the upper and lower parts of the Vistula River (Kraków and Kiezmark) and the Oder River (Chałupki and Krajnik) [4, 5, 7, 8].

The levels of radioactive contamination were higher in the samples collected in the upper parts of the rivers, at Kraków on the Vistula River and at Chałupki on the Oder River, than in the samples collected in the lower parts of the rivers. This agrees with the fact that southern Poland was contaminated more than northern Poland with radioactive fallout from the Chernobyl accident.

**Fig. 3:**
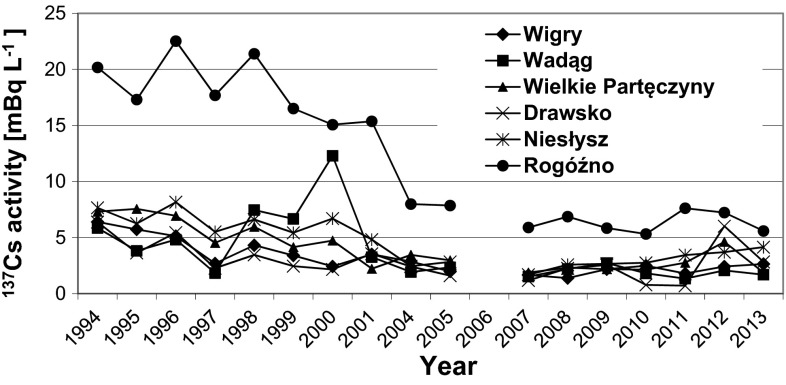
Annual average ^137^Cs activity concentration in water samples from the lakes that were studied

The plots in Fig. 3 show the ^137^Cs concentrations found in the lakes that were studied between 1994 and 2005 and between 2007 and 2013 [4, 7, 8]. The ^137^Cs concentration remained more constant over the years in the lakes than it did in the main rivers. Again, the exception was Rogóźno Lake, in which the ^137^Cs concentration decreased significantly over the study period.

**Fig. 4:**
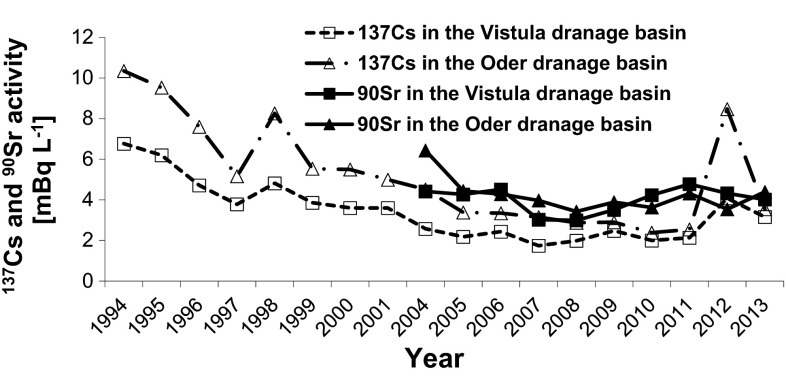
Annual average ^90^Sr and ^137^Cs activity concentrations in the Vistula and Oder drainage basins

It should be stressed that the first measurements of ^90^Sr contamination of water in Poland were performed in 1961. A number of nuclear explosions, especially the intense period of nuclear tests in 1951–1958 and 1961–1962, caused the ^90^Sr concentrations in the water samples collected in 1961 to have a very broad range, from 4 to 104 mBq L^−1^. The ^90^Sr concentrations in 1962 were 17–60 mBq L^−1^ . The mean ^90^Sr concentration in water in the Vistula estuary on the Baltic Sea was 10.2 ± 1.5 mBq L^−1^ in 1983 and the ^137^Cs concentration was only 1.0 ± 0.1 mBq L^−1^. Similar concentrations have been found in lakes in northern Poland, e.g., the ^90^Sr and ^137^Cs concentrations in water samples from Żarnowieckie Lake in the late 1970s were 9.58 ± 1.39 and 2.68 ± 0.21 mBq L^−1^, respectively [9]. Fallout connected with nuclear bomb explosions around the world between 1945 and 1980 is still the main source of ^90^Sr in the environment. Comparable amounts of ^90^Sr and ^137^Cs were present in the bomb-related fallout. Smaller amounts of ^90^Sr than ^137^Cs were released by the Chernobyl accident because Sr is not very volatile, and this means that the environment was affected by much less Sr contamination than Cs contamination. Therefore, the presence of ^90^Sr in the environment is mainly related to nuclear weapons testing in the atmosphere, and the reasons for the ^90^Sr variations that are observed in water bodies are rather complex [10]. The ^90^Sr and ^137^Cs concentrations in the samples from the rivers belonging to the Vistula and Oder drainage basins are shown in Fig. 4.

Large floods in the spring or early summer in 1997, 2007, and 2010 affected the ^137^Cs activity concentrations in the samples from the Vistula and Oder drainage basins. The surface water was diluted by rainwater particularly strongly in 1997. Large amounts of rainwater entered one or both of the drainage basins in those years (e.g., in 2010 the average water flow in the upper Oder River was 124 m^3^ s^−1^, while the average water flow in the upper Vistula River was only 43 m^3^ s^−1^) [11]. The ^137^Cs and ^90^Sr concentrations found in 1997, 2007, and 2010 were, therefore, lower than the concentrations that were found in the following years. This is particularly clear when the concentrations found in 1998 and 2012 are inspected (the river levels were low in 2012, and the average water flows in the upper Oder River and the upper Vistula River were 43.5 m^3^ s^−1^ and 14.9 m^3^  s^−1^, respectively). The average ^137^Cs concentration in the Vistula drainage basin in 1997 was 3.78 ± 0.31 mBq L^−1^, and the concentration had increased to 4.82 ± 0.42 mBq L^−1^ in 1998. The average ^137^Cs concentrations in the Oder drainage basin were 5.16 ± 0.49 mBq L^−1^ in 1997 and 8.26 ± 0.78 mBq L^−1^ in 1998. The ^137^Cs concentrations were 4.08 ± 0.21 mBq L^−1^ in the Vistula River and 8.47 ± 0.68 mBq L^−1^ in the Oder River in 2012, and these concentrations were very close to the concentrations found in 1998. This proves that the meteorological conditions have a direct impact on the concentrations of radioactive elements in water bodies.

The mean ^90^Sr activity concentrations found in samples from the Vistula and Oder Rivers have been consistent since 2004. Despite the relatively small input of ^90^Sr to the environment from the Chernobyl accident, the ^90^Sr concentrations were up to two times higher than the ^137^Cs concentrations in the river water samples from 2004 to 2011. The ^90^Sr and ^137^Cs concentrations in the river water samples were similar in 2013, indicating that ^90^Sr is still being washed out from the soil.

**Fig. 5:**
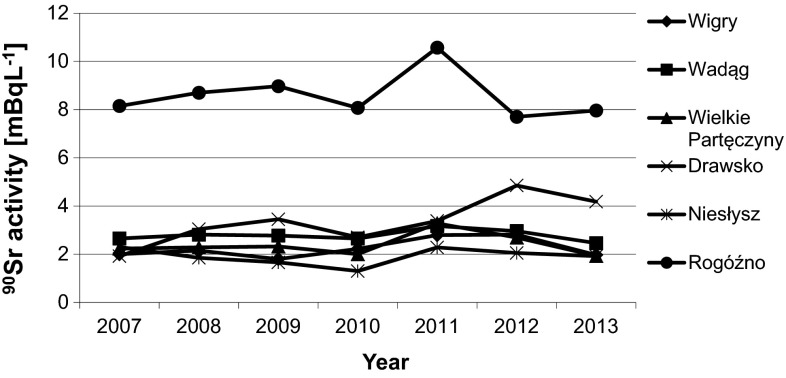
Annual average ^90^Sr activity concentration in the lakes that were studied

The ^90^Sr concentrations found in the lake water samples are presented in Fig. 5. As stated earlier, the highest concentrations were always found in the Rogóźno Lake samples. The concentrations in the other lakes were quite constant between 2007 and 2013. The ^90^Sr and ^137^Cs concentrations in the lake water samples were comparable.

The radionuclide concentrations in the Polish water bodies were fairly similar to the concentrations that have been found in rivers and lakes in other regions of Europe. For instance, the ^137^Cs concentrations found in Lugano Lake and the rivers that feed into the lake were around 1 mBq L^−1^ in 2003–2004 [12]. In 2012, the ^137^Cs concentrations found in four rivers in Finland were in the range 1.5 to 22 mBq L^−1^, and the ^90^Sr concentrations were in the range 2.5 to 6.4 mBq L^−1^ [13]. ^90^Sr concentrations of <1 to 6 mBq L^−1^ were found in the Netherlands in 2011 [14]. ^90^Sr concentrations of 1.2–4.3 mBq L^−1^ and ^137^Cs concentration of <1.5 mBq L^−1^ were found in three rivers in Portugal in 2011 [15].

## Conclusions

The ^137^Cs and ^90^Sr concentrations found in surface water samples from Poland in 2012 and 2013 were compared with the concentrations found in previous years, with the aim of identifying temporal changes. The ^137^Cs and ^90^Sr concentrations have clearly but rather slowly decreased over the years. The ^137^Cs mainly originated from the Chernobyl disaster, while the ^90^Sr is to a large extent the residue of nuclear weapons explosions in the atmosphere that were performed in the 1950s and 1960s. Despite the differences in the times that the ^137^Cs and ^90^Sr were released into the atmosphere, the ^137^Cs and ^90^Sr concentrations in the surface water samples were quite similar.
